# Nutritional Adequacy of Commercial Complementary Cereals in Germany

**DOI:** 10.3390/nu12061590

**Published:** 2020-05-29

**Authors:** Melissa A. Theurich, Berthold Koletzko, Veit Grote

**Affiliations:** Department of Pediatrics, Dr. von Hauner Children’s Hospital, University Hospital, LMU Munich, Lindwurmstraße 4, 80337 München, Germany; Melissa.Theurich@med.uni-muenchen.de (M.A.T.); Veit.Grote@med.uni-muenchen.de (V.G.)

**Keywords:** micronutrients, complementary feeding, complementary cereal, processed cereal based food, breakfast cereal, carbohydrates, sugar, Germany, Europe, infants and children

## Abstract

Commercial cereals are among the first complementary foods fed to infants in Germany and elsewhere. The purpose of this national survey is to describe the nutritional adequacy of commercial complementary cereals. A comprehensive, cross-sectional survey of cereal manufacturer websites (*n* = 15) was conducted from March to April 2019. Food labels were analyzed for iron, zinc, iodine, sodium, and sugar contents in commercial complementary cereals, and ingredient lists were evaluated for whole grains and added sugars. Preparation instructions were evaluated for the type of liquid recommended for reconstitution. Among 164 commercial complementary cereals, few contain iron (*n* = 43, 26%), zinc (*n* = 23, 14%) or iodine (*n* = 43, 26%). Sodium contents fall within EU thresholds. Most cereals were single grain, containing only wheat (*n* = 54), with half of the products (*n* = 86, 52%) containing whole grains. The average carbohydrate content of dry cereals is 69 g/100 g ± 9 g of which 14 ± 15 g is sugar. Preparation instructions for breakfast porridges and cereals recommend formula or toddler milk, while few recommend human milk (*n* = 13, 18%). Few commercial complementary cereals contain appreciable amounts (at least 15% of daily reference values) of zinc, iron, or iodine. A quarter of cereal carbohydrates are sugar and one-third of the products contain added sugars. Future directives should stipulate minimum micronutrient levels, strictly regulate sugar contents, and include human milk among preparation instructions.

## 1. Introduction

Commercial complementary foods (CCF), also known as industrial baby foods, contribute a large proportion of the diets of infants and toddlers in high-income countries. A recent study from the WHO European Regional office [1] reports high intakes of CCF across European countries, which coincides with studies from Germany [2,3,4].

Data from 3274 children enrolled in the Feeding Infants and Toddlers Study (FITS) in the United States reported that 51% of infants aged four to five months, 75% of infants aged six to eight months, and 52% of infants aged nine to eleven months consume commercial complementary cereals, also known as processed cereal based food (PCBF) [5]. In Europe, an analysis of dietary intakes of 1088 infants and children enrolled in the EU Childhood Obesity Project (CHOP) demonstrated that the most commonly consumed CCF were commercial cereals [2]. In the CHOP cohort, the median (IQR) daily energy intake from commercial cereals at six months of age was 69 kcal (25 to 126) per day, peaking at 126 kcal (73 to 177) per day at nine months of age, comprising about a third of caloric energy from all complementary foods at both time points and making up almost 100% of grain intakes at 6 months of age [2]. Commercial cereals were also the most commonly reported type of CCF at 4 months of age, demonstrating that they are among the first complementary foods introduced in European infants. An earlier analysis, of 688 German infants enrolled in the DONALD Study, showed 418 different varieties of CCF and infant formula on dietary records, of which 8% were infant formula, 22% were dry cereal-based foods, and 70% were ready-to-eat baby foods [4].

Suboptimal micronutrient intakes and micronutrient deficiencies are common among infants and young children in European countries [6,7,8]. Iron deficiency is more prevalent in certain groups, such as infants and children born with a low birth weight [9]. Iron deficiency has been shown to affect around 14% of one- to two-year-old children in other high-income countries such as the United States [5]. Iron depletion (serum ferritin <12 ng/mL) has also been reported amongst 10-month-old German infants [10]. Dietary data from infants and children enrolled in the CHOP trial in five European countries, including Germany, showed that intakes of iron and iodine, along with various other micronutrients, were inadequate [11].

Fortified cereals are often the primary type of complementary food providing non-heme iron for infants between six and 12 months of age [5] and are an important source of key micronutrients such as iron, zinc, and iodine. Given the popularity of commercial cereals in German cohorts, accumulating international evidence of inappropriate commercial complementary foods [1,12] and evidence of inadequate micronutrient intakes in Europe, an evaluation is warranted of the nutritional adequacy of commercial complementary cereals on the market in Germany.

Only limited empirical data is available on the nutritional quality of commercial complementary cereals in European countries. This cross-sectional survey of commercial complementary cereals gives a detailed and comprehensive evaluation of products sold in Germany. The primary objective of this study is to describe the nutritional adequacy of German commercial complementary cereals. The main findings include that many commercial cereals in Germany are poor sources of iron, zinc, and iodine, have too much sugar, contain added sugars, and lack preparation instructions with human milk.

## 2. Materials and Methods

In this cross-sectional survey of commercial cereals, ingredient and nutrient information was recorded from manufacturer and distributor websites between March and April 2019. The following baby food brands (*n* = 15) that make up almost all commercial baby cereals in Germany were included in the survey: Alete, Alnatura, BabyDream, BabyLove, Bebivita, dmBio, Hipp, Holle, Humana, Kölln, Löwenzahn Organics, Milasan, Milupa, Nestlé, and Töpfer.

A Microsoft Excel template was created for recording nutrient information from websites, and the product information was manually entered. The template was used to record information directly from digital food labels by a qualified nutritionist. Nutrient information recorded included total energy, total carbohydrates, total sugar, sodium, salt, and selected micronutrients (iron, zinc, and iodine). For dry commercial complementary cereals, nutrient information per 100 g of powdered product was recorded. For ready-to-eat commercial complementary cereals, nutrient information per 100 g of the product was recorded.

Information on the recommended age for consumption, ingredients list, and preparation instructions were also recorded. A second qualified nutritionist double checked the recorded values against manufacturer websites for potential recording errors.

Nutrient facts labels were searched to determine the level of sodium in the cereals. If the labels only reported grams of salt but not sodium, the level of sodium was calculated by assuming there was 0.4 g of sodium in one gram of salt. Sodium values reported for 100 g of prepared cereal were used. For products that only reported sodium levels for 100 g of dry cereal (*n* = 33), the level of sodium was calculated for prepared cereal. This calculation was based on the manufacturer recommended preparation instructions and a reference value for sodium in whole milk (0.1 g of salt per 100 mL). For products with multiple variations of possible cereal porridge recipes (using infant formula or a mixture of whole milk and water or other combination), sodium values for a mixture of cow’s milk (100 mL) and water (100 mL) were used (*Halbmilchbrei*). One manufacturer did not provide preparation instructions for three products. For these three products, a reference recipe was created using 25 g of cereal, 100 mL of cow’s milk, and 100 mL of water, a portion size similar to other cereals.

Ingredient lists were used to determine if commercial complementary cereals had added sugars and to quantify the total amount of milk ingredients by weight. Ingredient lists were searched by a qualified nutritionist for added sugars. Product ingredients that included added sucrose, honey, added fructose, chocolate, fruit juice concentrates, or vegetable juice concentrate were classified as sweetened. A second nutritionist checked the ingredients lists for recording and categorization errors. Cereals which contained unconcentrated fruit juices, dried fruit (i.e., raisins), fruit powders (i.e., apple powder), fruit flakes (i.e., banana flakes), and fruit extracts (i.e., apple extract) were not classified as sweetened. Fruit ingredients from ingredients lists were flagged to determine the total number of cereals containing fruit ingredients.

Nutrient content of commercial complementary cereals in Germany are regulated under the European Commission Directive 2006/125/EC regarding processed cereal-based foods and baby foods for infants and young children, in which processed cereal-based foods are divided into four main categories, namely [13]:

1. Simple cereals which are, or have to be reconstituted, with milk or other appropriate nutritious liquids;
2. Cereals with an added high protein food which are, or have to be reconstituted, with water or other protein free liquid;
3. Pastas which are to be used after cooking in boiling water or other appropriate liquids;
4. Rusks and biscuits which are to be used either directly, or after pulverization, with the addition of water, milk, or other suitable liquids.

Products in Categories 1 and 2 were included in the survey. Dry pasta and rusks (biscuits and cookies) were not evaluated, as the purpose of this survey was to evaluate the nutritional adequacy of commercial cereal porridges commonly used as first complementary foods. Infant formulas with added grains advertised as a beverage (*Trinkbrei*, *Trinkmahlzeiten, Gute Nacht Fläschen*) were not evaluated.

According to the EU labeling laws, information on vitamins and minerals must be expressed as a percentage of the reference values per 100 g or 100 mL of the product as sold [13]. Where appropriate, micronutrient information is also given per specified quantity of the prepared product, as it is recommended for consumption. Micronutrient levels are only reported on food labels when they are present in appreciable amounts (defined as at least equal to 15% of daily reference values) [13].

Descriptive and inferential statistics, tables, and figures were generated using Microsoft Excel. Student’s unpaired *t*-test was used to test differences between the mean caloric, carbohydrate, and sugar levels of sweetened and unsweetened cereals. The Chi-square test was used to test differences in the proportion of sweetened products by commercial cereal categories (grain porridges, milk porridges, and breakfast cereals).

## 3. Results

Nutrition and ingredient information was collected from 164 commercial cereal products from 15 brands. The following brands and number of respective cereal products, were included: Alete (*n* = 12), Alnatura (*n* = 14), BabyDream (*n* = 13), BabyLove (*n* = 5), Bebivita (*n* = 11), dmBio (*n* = 10), Hipp (*n* = 27), Holle (*n* = 13), Humana (*n* = 7), Kölln (*n* = 3), Löwenzahn Organics (*n* = 4), Milasan (*n* = 3), Milupa (*n* = 23), Nestlé (*n* = 6), and Töpfer (*n* = 12).

### 3.1. Grain Types

**Figure 1:**
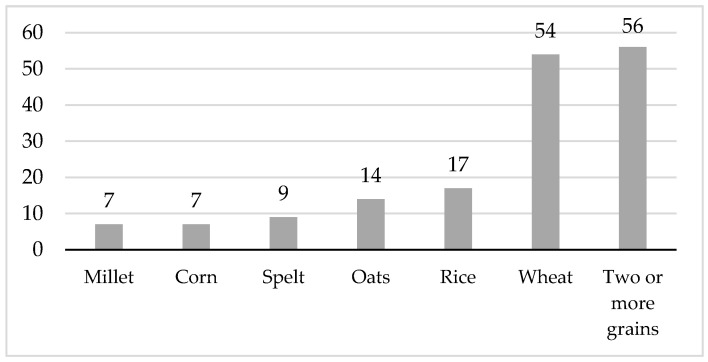
Number of commercial complementary cereal products by grain type.

The majority of commercial complementary cereals (*n* = 108, 66%) contained one type of the following grains: millet, corn, spelt, oats, rice, or wheat. One-third of the products (*n* = 56, 34%) contained two or more types of grains. Among the products containing a mixture of grains, the predominate type of grain by weight was wheat or oats. Half of the commercial complementary cereals evaluated (*n* = 86, 52%) contained at least one type of whole grain flakes or whole grain cereal flours. Figure 1 shows the number and percentage of complementary cereals by grain type.

The majority of cereals (*n* = 121, 74%) are labeled with advice to be fed from five (*n* = 61, 37%) or six months of age (*n* = 60, 37%) onwards. The remaining complementary cereals are advertised from seven (*n* = 1), eight (*n* = 15, 9%), 10 (*n* = 9, 5%), 12 (*n* = 18, 11%), or 15 months of age (*n* = 1) onwards.

### 3.2. Categories of Commercial Cereals

There were three main product categories: (1) milk porridges (*Milchbrei*), (2) grain porridges (*Getreidebrei*), and (3) breakfast cereals and porridges (*Kinder-Müsli, Kinderporridge*). There were two main types of cereals. The two types of cereals were a) dry commercial complementary cereals requiring reconstitution with liquids (*n* = 132, 80%) and b) ready-to-eat products sold in baby food jars, tubs or pouches (*n* = 33, 20%).

Milk porridges are cereal porridges with milk ingredients which are often advertised as “evening porridges” (*Abendbrei*) or “goodnight-porridges” (*Gute Nacht Brei*). Infant formula, as well as skimmed and whole animal milk powders comprise a large range of the product weight in milk porridges. Some products included percent weight of follow-on infant formula as a single ingredient in the ingredients list while other products listed individual ingredients found in formula (skimmed milk powder, plant oils, vitamins, etc.). Amongst the dry milk porridges that listed follow-on infant formula as an ingredient (*n* = 24), the percent weight for follow-on formula was on average 34% ± 10% of product weight. Among the dry milk porridges with several listed ingredients (*n* = 36), skimmed milk powder comprised an average of 18% ± 6% of product weight. In addition, some milk porridges contained whey powder (*n* = 46). Among the ready-to-eat milk porridges listing skimmed (*n* = 13) or whole milk (*n* = 20) as ingredients, milk comprised an average of 68% ± 22% and 54% ± 22% of product weight, respectively.

Grain porridges are commercial complementary cereals without animal milk components. These porridges sometimes contain fruit (*Getreide-Obst Brei*). Products in this category are marketed for infants starting from 5 months of age onwards.

Breakfast cereals and porridges for young children (*Kindermüsli, Kinderporridge, Juniormüsli*) do not contain animal milk components but can contain dried fruit. Products in this category are marketed for infants and young children 10 months of age and older.

One-third of all commercial cereals contained fruit (not including products containing only fruit juice). Banana was by far the most popular type of fruit ingredient (43 of 47 fruit-containing cereals, 91%), in the form of banana puree, banana flakes, or banana powder. Fruit was an ingredient across all three product categories.

### 3.3. Preparation Instructions

Due to the inclusion of dry milk ingredients, preparation instructions for dry milk porridges require reconstitution with water only. Instructions for dry grain porridges include reconstitution with various other types of liquids, including a mixture of 50% whole cow’s milk (3.5–3.8% fat) and 50% water (*Halbmilchbrei*), infant formula, follow-on formula, or human milk. Some manufacturers give “dairy-free” preparation instructions which include reconstitution with a combination of water, fruit puree, and vegetable oils. Of all dry grain porridges, only two manufacturers give instructions for reconstitution of dry cereals with human milk for breastfed infants (*n* = 13, 24%), whereas most give instructions for reconstitution with infant formula or toddler milk (infant formula marketed for young children) (*n* = 35, 65%).

Breakfast cereals and porridges are recommended by manufacturers to be prepared with various milk products. Preparation instructions for breakfast cereals included instructions for reconstitution with whole cow’s milk (*n* = 4, 24%). Ten products (*n* = 10, 59%) recommended preparation with toddler milk. Three brands recommending preparation of cereals with formula for young children promoted their own formula brand. Breakfast cereals marketed from 10 months of age onwards (*n* = 3) included instructions for reconstitution with infant formula, cow’s milk, or a “dairy-free” preparation with pureed fruit and vegetable oils. There were no breakfast cereals marketed from 10, 12 or 15 months of age that included preparation instructions with human milk.

### 3.4. Key Micronutrients

Micronutrient contents of commercial complementary cereals vary by whether cereal products were dry or ready-to-eat. According to food labels, none of the ready-to-eat products contain iron, zinc, or iodine in appreciable amounts, defined as at least equal to 15% of daily reference values.

Less than one-third of all commercial complementary cereals surveyed report the iron content on their nutrient labels (see Table 1). Most products containing iron are recommended for infants starting from five or six months of age onwards. The majority of cereals that report iron on the food label are fortified with ferric diphosphate (ferric pyrophosphate) (*n* = 35), ferrous sulfate (*n* = six), or ferrous fumarate (*n* = one).

Few cereal products surveyed report zinc on nutrient labels (see Table 1). The majority of products containing zinc are recommended for infants from five or six months of age onwards. All commercial complementary cereals containing zinc were fortified with zinc sulfate (*n* = 10) or zinc gluconate (*n* = 4).

One-third of the products surveyed reported iodine on nutrient labels (see Table 1). Most products containing iodine were recommended for infants starting from five or six months of age onwards. Products containing iodine were fortified with potassium iodide (*n* = 18, 42%) or potassium iodate (*n* = 21, 48%). Three commercial cereals reported iodine on nutrient labels but did not list the source of iodine. These cereals reported follow-on formula in the ingredients list, a dietary source of iodine, according to the EU regulations which mandate between 10–50 µg iodine/100 kcal of formula. The iodine content from one cereal product, which was not fortified and did not contain follow-on formula, could not be verified by the ingredients label.

### 3.5. Sodium

The average sodium content of all dry commercial complementary cereals, when reconstituted as instructed by the manufacturer, was 27 ± 7.0 mg/100 kcal. The sodium content of ready-to-eat cereals was 39 ± 11 mg/100 kcal.

### 3.6. Carbohydrates and Sugars

The average carbohydrate content of dry commercial cereals was 69 ± 9 g/100 g, with 14 ± 15 g from total sugar. Most sweetened cereals products contained sucrose or fruit juice concentrates. Table 2 shows the number of sweetened products by the type of added sugar.

**Figure 2:**
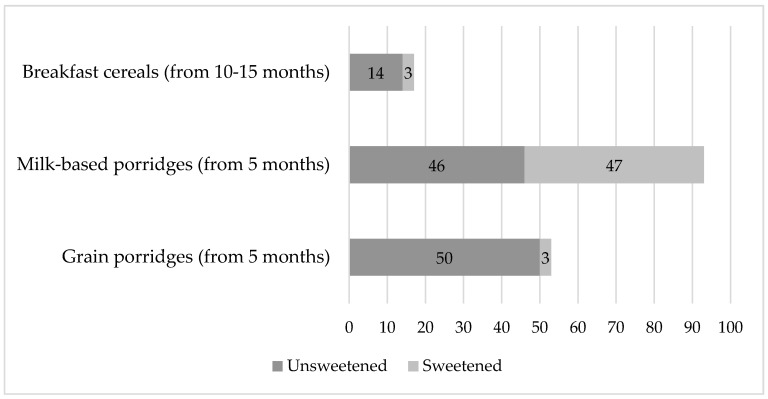
Proportion of sweetened commercial cereals by category.

One-third of the commercial cereals evaluated (*n* = 52, 32%) contained added sugars. The milk porridges category had significantly more sweetened products (*p <* 0.001) as compared with grain porridges and breakfast cereal categories. Figure 2 shows the proportion of sweetened products in each product category.

Total energy and carbohydrate contents of commercial complementary cereals differed depending on whether cereals had been sweetened with added sugars. Table 3 shows the mean energy, as well as carbohydrate and sugar contents of sweetened and unsweetened dry cereals (*n* = 80).

A comparison with unsweetened dry cereals showed that sweetened dry cereals provided, on average, significantly more calories (*p* < 0.001, mean difference = 21 kcal/100 g), more carbohydrates (mean difference = 1.8 g/100 g), and significantly more sugar (*p* < 0.001, mean difference = 5.5 g/100 g). 

Sweetened ready-to-eat cereals provided significantly more carbohydrates (*p* < 0.001) and sugar (*p* < 0.001) than unsweetened ready-to-eat cereals. Table 4 shows the mean energy, carbohydrate and sugar contents by cereal category (sweetened or unsweetened) for ready-to-eat cereals (*n* = 33).

## 4. Discussion

### 4.1. Preparation Instructions

The nutritional value of dry grain cereals depends on the liquids used to reconstitute them. We found a wide range of liquids recommended for reconstitution in the manufacturers’ preparation instructions for commercial complementary cereals. Recipes for homemade complementary cereals in Germany advise, as one option, to include up to 200 mL/day of cow’s milk, whereas human milk or infant formula are also indicated as options to prepare cereals [14]. Of note, commercial milk porridges, in contrast to homemade milk porridges, cannot be prepared with human milk because they contain dried whole or skimmed animal milk or infant formula, and therefore require reconstitution with water only.

### 4.2. Key Micronutrients

German national infant feeding advice [15] recommends introducing foods high in critical micronutrients such as iron, zinc, and iodine as first complementary foods. A blend of vegetables, potato, and meat or fish (*Gemüse-Kartoffel-Fleischbrei*) is recommended as a first complementary food because of its high content of bioavailable micronutrients [15]. Subsequently, milk porridges should be introduced starting from five months of age (20 weeks) and fruit-grain porridges (*Getreide-Obst Brei*) are recommended from six months (24 weeks) of age [15].

Iron, zinc, and iodine are important for infant health and complementary foods should be good sources of these micronutrients to ensure adequate growth and development. During the first years of life, dietary iron is important for infants’ neurological and cognitive development. Commercial complementary cereals are considered to be important non-heme sources of iron for infants during the complementary feeding period [5]. To ensure sufficient intakes of iron in infancy, fortified complementary cereals are useful for the provision of iron. In our study, commercial complementary cereals with the highest amounts of iron were fortified. However, we found that the majority of commercial complementary cereals sold in Germany are not fortified and are poor dietary sources of iron (containing less than 15% of recommended daily intakes). Similarly, commercial baby food jars evaluated in Spain that contained meat, fish, vegetables and fruit, and also had low iron contents contributing only about 5–20% of adequate intakes [16]. Data from 3274 children enrolled in the Feeding Infants and Toddlers Study (FITS) in the United States showed that infants and toddlers who consumed fortified commercial complementary cereals had higher iron intakes as compared with non-consumers [5].

The European Commission issued a directive in 2006 that established maximum, but not minimum levels, for iron, zinc, and iodine in commercial complementary cereals [13]. The Codex Alimentarius Guideline on Formulated Complementary Foods for Older Infants and Young Children [17] gives reference values as a guide for the amounts of vitamins and minerals to be added to complementary foods, including cereal-based porridges. For infants aged six to 12 months with an average body weight of 9 kg, the WHO and FAO-recommended nutrition intake (RNI) for iron is 18.6 mg and 9.3 mg at 5% and 10% dietary iron bioavailability, respectively [18]. For children aged 12 months to three years with an average body weight of 13 kg, the RNI is 11.6 mg and 5.8 mg at 5% and 10% dietary iron bioavailability, respectively [18]. Therefore, the WHO and FAO suggested levels of iron contained in a daily ration of complementary cereal should be 4.7–9.3 mg for older infants, and 2.9–5.8 mg for young children [18]. Among all the cereals included in this survey, only 26% provided at least 15% of the RDI for iron. Manufacturers should ensure that commercial cereals are good sources of iron.

Zinc is important for adequate development of an infant’s immune system. Fortified cereals can contribute to an adequate zinc intake. A randomized study of 45 five-month-old breastfed infants in the United States demonstrated that zinc requirements are unlikely to be met without regular consumption of meat or zinc-fortified foods [19]. For infants aged six months to one year, the average individual normative requirements are 0.3 mg/kg/d and 0.186 mg/kg/d at moderate (30%) and high (50%) zinc bioavailability, respectively [18]. For children aged one to three years, the average individual normative requirements are 0.23 mg and 0.14 mg at moderate and high zinc bioavailability, respectively [18]. Therefore, the WHO and FAO suggested level of zinc contained in a daily ration of complementary cereal should be 0.16–0.93 mg for infants and 0.12–0.69 mg for young children. However, according to results of this survey, only 14% of commercial complementary cereals in Germany provided at least 15% of the RDI for zinc. Manufacturers should ensure that commercial cereals are good sources of zinc.

During the first years of life, iodine is important for the development of the thyroid and central nervous system. To ensure sufficient iodine intake, consumption of iodine-fortified complementary foods is recommended [20]. According to the WHO and FAO guidance [18], infants from birth to three years of age, the daily iodine intake recommendation is 90 µg/day or 6–30 µg/kg/day. The suggested total quantity of iodine contained in a daily ration of complementary cereal should be at least 50% of 90 µg/day [17], or 45 µg of iodine. In our study, dry cereals containing iodine had a median level of 59 µg/100 g dry product (IQR 50–104) and only 28% of commercial complementary cereals surveyed provided at least 15% of the RDI for iodine.

The authors of a market survey on CCF in Germany, in 2008, reported a higher proportion of commercial cereals fortified with iodine as compared with this survey. In that survey, 80 (83.3%) of 98 milk porridges surveyed were fortified with iodine, and the median iodine level of fortified milk porridges was 21 µg/100 g (IQR 8, 29) for ready-to-eat products [20]. This is presumably because milk porridges contained infant formula which had been fortified with iodine. However, only 6 of 45 (13.3%) fruit-cereal porridges were fortified with iodine, with a median iodine concentration of 20 µg/100 g (IQR 6, 20) for ready-to-eat products. Researchers also modeled dietary intakes of an 8-month-old infant fed one of three daily diets consisting of either human milk (with and without maternal iodine supplementation), or fortified infant or follow-on formula. Complementary meals in the modeled diet consisted of either homemade or fortified commercial complementary food. The results showed that a breast-fed infant getting homemade porridges obtained less than 50% of the recommended iodine intake [20]. An infant diet modeled using infant formula and fortified commercial porridges, exceeded recommended intakes by 39–100%, depending on the products chosen [20]. The authors concluded that fortification of commercial complementary cereals is necessary to ensure adequate iodine intakes, especially for breastfed infants [20]. Our survey demonstrates that very few commercial complementary cereals in Germany are fortified with iodine. Manufacturers should ensure that commercial cereals are good sources of iodine to supply adequate iodine for all infants, especially breastfed infants.

### 4.3. Sodium

Sodium salts can only be added to processed cereal-based baby foods for technological purposes [13]. Diets high in salt have been associated with non-communicable diseases such as hypertension, cardiovascular diseases, stomach cancers, and chronic kidney disease. In Germany, the intake of salt in the population is estimated using data from the German Health Interview and Examination Survey for Adults (DEGS), from 2008 to 2011. For infant girls and boys aged six months to one year, the median daily salt intake is 1.1 g and 1.4 g, respectively [21]. Studies from the United States have shown some commercial complementary foods to be high in salt [22]. According to the European directive from 2006 [13], sodium content for cereals shall not exceed 100 mg/100 kcal for ready-to use products or dry cereals when reconstituted as instructed by the manufacturer. Results from this survey did not show commercial complementary cereals in Germany to exceed the maximum level given in the 2006 EU Commission Directive [13]. In 2019, the WHO Europe recommended to further reduce the total sodium in CCF to 50 mg/100 kcal for most products [12].

### 4.4. Total Carbohydrates, Total Sugar, and Added Sugars

Many commercial cereal products surveyed contained high levels of sugar and added sugars (sucrose, glucose, honey, and fruit juice concentrates). Approximately one-third of the commercial cereals contained fruit, mostly from ingredients containing banana. A study from the United Kingdom evaluated the types of fruits and vegetables used in 329 CCF which had the fruits and vegetables in the product name and reported that CCF contained predominantly fruits and relatively sweet vegetables [23]. This is of concern, since high sugar intakes can contribute to the risk of childhood overweight/obesity and dental caries [24]. Furthermore, exposure to sweet products during infancy can promote a preference for sweet foods [25] and poor eating habits in childhood [26].

The 2006 EU Commission Directive set maximum levels for added sugars such as sucrose, fructose, glucose, glucose syrups, and honey in dry commercial complementary cereals [13]. The amount of added carbohydrates from all of these sources should not exceed 7.5 g/100 kcal, and should not exceed 3.75 g/100 kcal for added fructose [13]. However, manufacturers are not required by current EU labeling laws to report the quantity or percent weight of added sugars. Therefore, currently consumers can only draw conclusions on the addition of sugar from the ingredients list, while it is not possible to quantify added sugar.

A European Union report based on data in the Mintel GNPD database, published in 2019, included 4196 infant foods and 502 different processed cereal-based foods [27]. This report showed that 1359 (31.9%) baby foods had added or free sugars and 1167 (27.4%) had one or more types of sugar among the top five ingredients [27]. Sugars were added predominantly (75% of products) to baby biscuits and rusks. The report included 483 products from Germany, of which only 53 products were dry commercial complementary cereals [27]. According to that report, the average energy of dry commercial complementary cereals from Germany was 386 kcal/100 g, with an average of 69.1 g of carbohydrates and 15.3 g total sugar [27]. These values are similar to the nutrient contents found in this study for dry cereals, with an energy content of 396 ± 32 kcal, 69.8 ± 6 g of carbohydrates and 17 ± 15 g sugar. German ready-to-eat commercial cereal products were not included in the EU report.

In 2019, the WHO Regional Office for Europe published a report on 7955 CCF and drink products in Vienna, Austria, Sofia, Bulgaria, Budapest, Hungary, and Haifa, Israel [1]. This report included information from both dry and ready-to-eat commercial cereals and showed that around one-third of dry cereals containing whey or milk powder contained a 30% mean percentage of energy from total sugar (ranging from 29% in Italy to 44% in Hungary) [12]. The results of this study showed similar values for milk porridges in Germany, with an average of 29.8% ± 7% of energy derived from sugar.

In 2019, WHO Europe called for complete prohibition of added sugars and sweeteners (including syrups, honey, fruit juice, fruit juice concentrates, and non-sugar sweeteners) in all commercial complementary foods [28]. In addition, WHO Europe is drafting a nutrient profile model to guide decisions about which foods are inappropriate for promotion for infants and young children six to 36 months of age [12]. The model has been validated against nutrient label information from 1328 products on the market in Denmark, Spain, and the United Kingdom and pilot tested on a further 1314 products from seven additional countries (Estonia, Hungary, Italy, Malta, Norway, Portugal, and Slovenia) [12].

## 5. Limitations

This survey consolidated nutrient information for commercial complementary cereals from food labels. Laboratory analysis of commercial complementary cereals would provide a more accurate assessment of actual nutrient contents. A laboratory analysis of 100 samples of CCF from the United States demonstrated that nutrient label data both under- and overestimated total sugars [29]. Approximately 25% of all foods evaluated had total sugar values with either less than 10% or more than 10% of total sugar contents listed on ingredients labels [29]. It seems possible that total sugar reported on food labels in Europe could also be different from actual sugar content.

Current EU food labeling hinders the evaluation of calories from added sugars in commercial complementary cereals. Lactose in milk porridges and fructose from fruit ingredients both contribute to total sugar content. Since sugars which naturally occur in milk and fruit are not currently differentiated from added sugars on CCF food labels, it was not possible to obtain information on the contribution of added sugars to total calories or total carbohydrates. In 2019, a policy brief from the WHO Regional Office for Europe proposed improvements of product labeling for sugar and total fruit content of CCF marketed in Europe [28].

A limitation of this cross-sectional study is that the available commercial food products, and potentially the nutritional composition of these products, are constantly changing. This has potential implications for reproducibility of this study. Multiple cross-sectional studies are needed to understand potential time-related trends in nutrient composition of commercial baby foods.

Sodium contents of prepared commercial cereals were based on food label sodium values and recipe simulation with a water and cow’s milk mixture (*Halbmilchbrei*). Without detailed data on actual preparation practices, it is not possible to fully reflect real-world scenarios.

## 6. Recommendations

Commercial complementary cereals are commonly consumed and often recommended as first complementary foods amongst German infants. Most of the commercial complementary cereals evaluated in this study were poor sources of iron, zinc, and iodine. One-third of the products contained added sugars. Few products recommended human milk for reconstitution. Nutrient composition of commercial complementary cereals should be improved, and regulatory standards should provide stronger guidance for an adequate composition and reconstitution that serves to promote child health.

## Figures and Tables

**Table 1:** Mean and median iron, zinc, and iodine contents of commercial complementary cereals as reported on food labels ^a^.

Age	Products	Iron			Zinc			Iodine		
Month	*n* (%)	*n*	Mean ^f^ (SD) ^b^	Median ^g^ (Q1, Q3) ^b^	*n*	Mean ^f^ (SD) ^c^	Median ^g^ (Q1, Q3) ^c^	*n*	Mean ^f^ (SD) ^d^	Median ^g^ (Q1, Q3) ^d^
5	61 (37)	13	5.5 (2.5)	6.5 (3.5, 7.3)	8	3.6 (1.2)	3.4 (2.7, 4.0)	13	68 (24)	62 (50, 70)
6	59 (36)	24	4.9 (2.3)	4.6 (0.1, 8.5)	11	3.1 (1.0)	2.8 (2.6, 3.0)	23	70 (27)	57 (50, 104)
7	1 (0)	1	0	0	1	0	0	1	0	0
8	15 (9)	7	5.5 (1.9)	5.3 (4.2, 7.3)	2	2.6 (0.2)	2.6 (2.4, 2.6)	7	76 (40)	65 (54, 110)
10	9 (5)	1	0	0	0	0	0	1	0	0
12	18 (11)	1	0	0	1	0	0	1	0	0
15	1 (0)	0	0	0	0	0	0	0	0	0
Total	164 (100)	47	5.4 (2.3)	5.3 (3.5, 7.3)	23	3.2 (1.0)	2.8 (2.6, 3.4)	46	70 (28)	59 (50, 104)

^a^ includes intrinsic and added iron, zinc, and iodine; ^b^ mg iron per 100 g dry product; ^c^ mg zinc per 100 g dry product ^d^ µg iodine per 100 g dry product; ^f^ mean of products reporting iron, zinc, or iodine on the food label; ^g^ median of products reporting iron, zinc, or iodine on the food label.

**Table 2:** Number of sweetened cereals by type of added sugar as reported on food labels.

Type of Added Sugar	Number of Products *n* (%)
Sucrose	30 (58)
Fruit juice concentrates	6 (12)
Chocolate powder (cacao, sucrose)	5 (10)
Vegetable juice concentrates	4 (8)
Fructose	4 (8)
Glucose	2 (4)
Honey	1 (2)
Total	52 (100)

**Table 3:** Mean energy, as well as carbohydrate and sugar contents of sweetened and unsweetened dry cereals as reported on food labels.

Dry Cereals	Total Products *n* (%)	Energy (kcal) Per 100 g Powder (Mean ± SD)	Carbohydrates (g) Per 100 g Powder (Mean ± SD)	Total Sugar (g) Per 100 g Powder (Mean ± SD)
sweetened	28 (17)	415 ± 23	70 ± 3	29 ± 10
unsweetened	103 (63)	393 ± 33	69 ± 9	15 ± 15

**Table 4:** Mean energy, as well as carbohydrate and sugar contents of sweetened and unsweetened ready-to-eat cereals, as reported on food labels.

Ready-to-Eat Cereals	Total Products *n* (%)	Energy (kcal) Per Portion (100 g) (Mean ± SD)	Carbohydrates (g) Per Portion (Mean ± SD)	Total Sugar (g) Per 100 g Portion (Mean ± SD)
sweetened	24 (15)	79 ± 7	12 ± 1	7 ± 1
unsweetened	9 (5)	65 ± 29	8 ± 4	3 ± 1
